# The Thoracic Anatomy of Two Flightless *Chrysolina* Species (Coleoptera: Chrysomelidae)

**DOI:** 10.3390/insects16060618

**Published:** 2025-06-11

**Authors:** Sipei Liu, Xiaokun Liu, Xieshuang Wang, Wenjie Li, Xin Liu, Siqin Ge

**Affiliations:** 1 State Key Laboratory of Animal Biodiversity Conservation and Integrated Pest Management, Institute of Zoology, Chinese Academy of Sciences, Beijing 100101, China; spliu@ioz.ac.cn (S.L.); liuxiaokun22@ioz.ac.cn (X.L.); 18801161767@163.com (X.W.); liwenjie@ioz.ac.cn (W.L.); 2 University of Chinese Academy of Sciences, Beijing 100101, China; 3 Department of Medical Research, The Ninth Medical Center, Chinese PLA General Hospital, Beijing 100101, China

**Keywords:** flightlessness, *Chrysolina*, thoracic morphology, light microscopy, SEM, 3D reconstruction

## Abstract

Flight loss has independently evolved across nearly all winged insect orders. Comparing the thoracic structures of flightless insects with those of their flight-capable relatives can reveal key characteristics linked with flight. Although flight loss has been widely studied in beetles, exploration of this phenomenon has been limited to taxonomic and geographic distribution studies in the species-rich family Chrysomelidae, with little analysis of thoracic anatomical structures. This study employs a suite of morphological techniques to examine the thoracic structures of two flightless beetle species *Chrysolina*: *sulcicollis* and *Chrysolina virgata*, originating from desert and temperate regions, respectively. A comparison between the two flightless species reveals that *C. sulcicollis* has fewer tergo-pleural muscles involved in elytral movement likely to save water, but more muscles that contribute to stabilizing larger body structures. Meanwhile, differences are also observed in the elytral base, the anterior corner of the mesal suture, and the setae on the meso-inner region of the epipleuron. Compared to other flight-capable chrysomelid beetles, apart from the absence of flight-related muscles, the two flightless beetles exhibit similar thoracic skeletal structures. The absence of lateral cervical sclerites, along with the presence of muscles Idvm4, 5 and Itpm5, could enhance head mobility as a compensatory adaptation doe the loss of flight capability. Additionally, the greater number of tergo-pleural muscles in the mesothorax of *C. virgata* could suggest that its elytra serve a specialized function. Compared to other flightless beetles, aside from the similarly reduced flight muscles, these two species have relatively intact thoracic skeletons. Further data on habitat, functional compensation and other related factors are needed to compare their evolutionary processes with those of other flightless beetles.

## Simple Summary

Flight loss has independently occurred in almost all winged insect groups. Studying the thoracic structures of flightless insects and comparing them to their flying relatives can help identify traits linked to flight. In beetles, especially the diverse Chrysomelidae family, most research has focused on taxonomy and distribution, with little attention to thoracic anatomy. This study compares the thoracic structures of two flightless *Chrysolina* species—*C. sulcicollis* from a desert region and *C. virgata* from a temperate region—using various morphological methods. Both species show reduced flight muscles and lack of cervical sclerites, but differ in elytral base structure, mesal suture shape, and epipleuron setae. *C. sulcicollis* has fewer elytra-controlling muscles and more muscles for body stabilization, likely due to water-saving needs. Compared to other flight-capable chrysomelid beetles, the two flightless species have similar thoracic skeletons, except for the missing flight-related muscles. The absence of cervical sclerites, combined with the presence of certain neck-related muscles, might help flightless beetles move their heads more freely, potentially compensating for their inability to fly. Also, the higher number of tergo-pleural muscles in the mesothorax of *C. virgata* could suggest that its elytra have a special function. Better understanding their evolutionary paths requires more data on environmental factors and the trade-offs linked to flight loss.

## 1. Introduction

Flight is a key evolutionary innovation that emerged in the early Carboniferous, significantly boosting both the diversity and abundance of insects [1]. However, flight loss has independently occurred in nearly all orders of Pterygota, driven by various evolutionary pressures [2,3]. The loss of flight might facilitate allopatric differentiation, potentially leading to high speciation rates in flightless lineages [4], which explains why flightless species account for approximately 10% of all described insect species [2]. Furthermore, wing reduction is commonly regarded as an evolutionary adaptation that enhances fecundity and increases survival probability, because it permits greater allocation of resources to traits that enhance environmental adaptation, such as higher oviposition rates or improved thermoregulation [2,3,5]. The insect thorax, which serves as the central support structure bearing two pairs of wings and three pairs of legs, is crucial to flight performance. For decades, comparisons between the thoracic structures of flightless insects and their flight-capable relatives have been studied to identify key features linked with flight and to infer the morphological mechanisms underlying insect flight, such as grasshopper, fruitfly and moth (e.g., [6,7,8]).

Most beetles have their forewings hardened into elytra, with the hindwing modified to fold beneath them. The hindwings are considered the primary source of propulsion during flight, while the elytra function mainly as protective coverings. Many studies have examined flightless beetles and revealed structural modifications in their metathorax [9,10,11,12,13,14]. These flightless beetles might occupy specialized habitats (e.g., caves, anthills) or display unique behaviors, such as death-feigning. Leaf beetles (Coleoptera: Chrysomelidae) comprise a group comprising over 37,000 species worldwide [15]. Previous research on flightless leaf beetles has primarily focused on taxonomy [16,17,18,19,20] and geographic distribution [21,22], while morphological studies have been largely limited to external features and male genitalia. Detailed anatomical investigations of the thorax—closely linked to flight capability—remain scarce. The current study we use various morphological techniques involved in light microscopy, scanning electron microscope (SEM), micro-CT and 3D reconstruction to study the thoracic structures of two flightless species of *Chrysolina*: *Chrysolina (Chrysocrosita) sulcicollis przewalskyi* (Jakobson, 1898) [23] and *Chrysolina (Euchrysolina) virgata* (Motschulsky, 1860) [24]. *C. sulcicollis* is distributed across Xinjiang, Gansu, Ningxia, and Shanxi in China, inhabiting arid desert environments. In contrast, *C. virgata* occupies temperate habitats in northern regions [15]. Field observations revealed that the elytra of *C. sulcicollis* are tightly locked, while those of *C. virgata* remain functional and can be opened. We hope this study provides additional evidence to further understand flight loss in insects.

## 2. Materials and Methods

The specimens from two species were used: *Chrysolina (Chrysocrosita) sulcicollis przewalskyi* (Jakobson, 1898) collected from Lishan Nature Reserve (altitude: 1200 m), Shanxi, China in March 2012, and *Chrysolina (Euchrysolina) virgata* (Motschulsky, 1860) collected from Badaling Forest Farm, Beijing, China in June 2017. The specimens were preserved in 70% ethanol.

The specimens were firstly cleaned using an ultrasonic cleaner (KQ-50DE, IZCAS, Beijing, China) for about 20 min. The specimens were then dehydrated with ethanol concentrations gradually increased from 70% to 100% in 5% increments and subsequently dried with a critical point dryer (Leica EM CPD 300, IZCAS, Beijing, China).

The overall images of the specimens were captured using a SLR camera (Canon 5D II, IZCAS, Beijing, China). Another SLR camera (Nikon D500, IZCAS, Beijing, China) connected with a stereoscope (Zeiss stereo Discovery V12, IZCAS, Beijing, China) was used to record detailed images of the thoracic structures. For each shot, 25 to 35 sequentially zoomed-in images were captured and stacked using Helicon Focus 2.0 (Helicon Soft Ltd., Kharkiv, Ukraine).

Some specimens were coated with golden powders with a sputter coater (Leica EM SCD 050, IZCAS, Beijing, China), attached to a circular metal plate using conductive adhesive and examined with scanning electron microscopy (FEI Quanta 450, IZCAS, Beijing, China).

The other specimens were pasted on the base of a pipette tip, with its tip end securely fixed onto the specimen holder, and scanned using micro-CT (Zeiss microXCT-400, IZCAS, Beijing, China) with a 4X objective lens at a beam strength of 40 kV. The thoracic structures of the two species were reconstructed based on an aligned image stack from micro-CT with Amira 6.0 (Thermo Fisher Scientific, Waltham, MA, USA). The 3D reconstruction results were exported as stacks of tiff files into VG Studio Max 3.0 (Volume Graphics, Heidelberg, Germany) for volume rendering.

Finally, all the figures from cameras, SEM and micro-CT were edited with Adobe Photoshop 2017 (Adobe Inc., Mountain View, CA, USA) and Adobe Illustrator 2017 (Adobe Inc., Mountain View, CA, USA) for layout and adding labels.

The terminology for the thoracic skeleton follows Ruan et al. [25] and Friedrich and Beutel [26] and for the musculature Friedrich and Beutel [27].

## 3. Results

### 3.1. Chrysolina (Chrysocrosita) sulcisollis przewalskyi (Jakobson, 1898)

**Figure 1:**
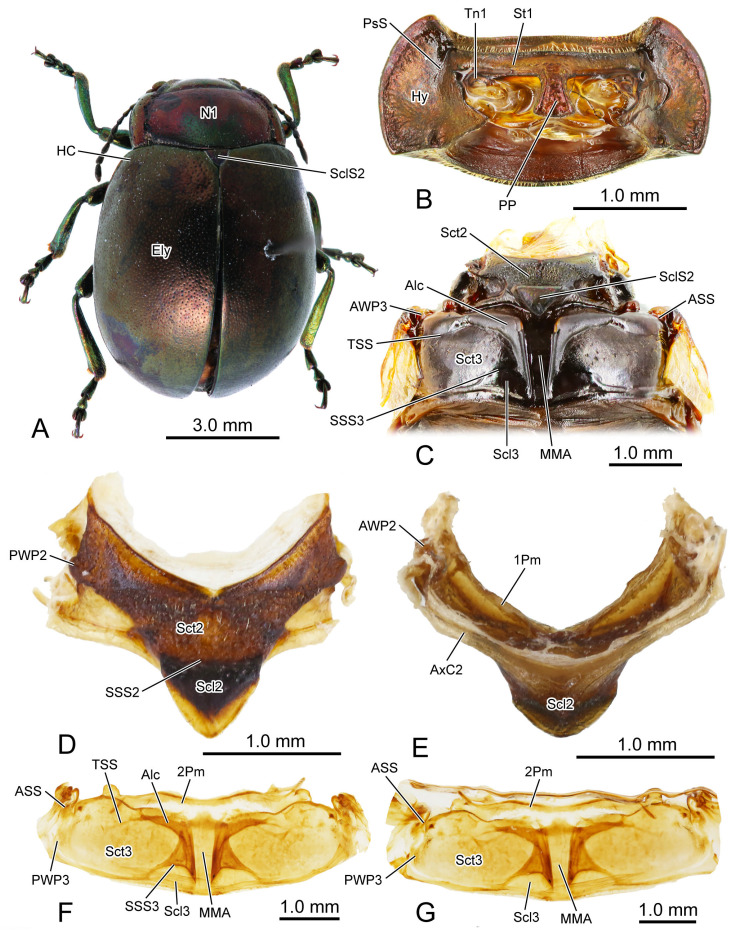
Digital photography of *Chrysolina sulcisollis*. (**A**) habitus, dorsal view; (**B**) prothorax, ventral view; (**C**) pterothorax, dorsal view; (**D**) mesothoracic tergite, dorsal view. (**E**) mesothoracic tergite, ventral view. (**F**). metathoracic tergite, dorsal view. (**G**) metathoracic tergite, ventral view. Abbreviations: 1/2Pm—pro-/mesophragma; Alc—alacrista; ASS—antero-lateral scutal suture; AWP2/3—anterior notal wing process of meso-/metathorax; AxC2—axillary cord of mesothorax; Ely—elytron; HC—humeral callus; Hy—hypomeron; MMA—median membranous area; N1—pronotum; PP—prosternal process; PsS—notosternal suture; PWP2/3—posterior notal wing process of meso-/metathorax; Scl2/3—meso-/metascutellum; SclS2—mesoscutellar shield; Sct2/3—meso-/metascutum; SSS2/3—scutoscutellar suture of meso-/metathorax; St1—prosternum; Tn1—protrochantin; TSS—transverse scutal suture.

**Figure 2:**
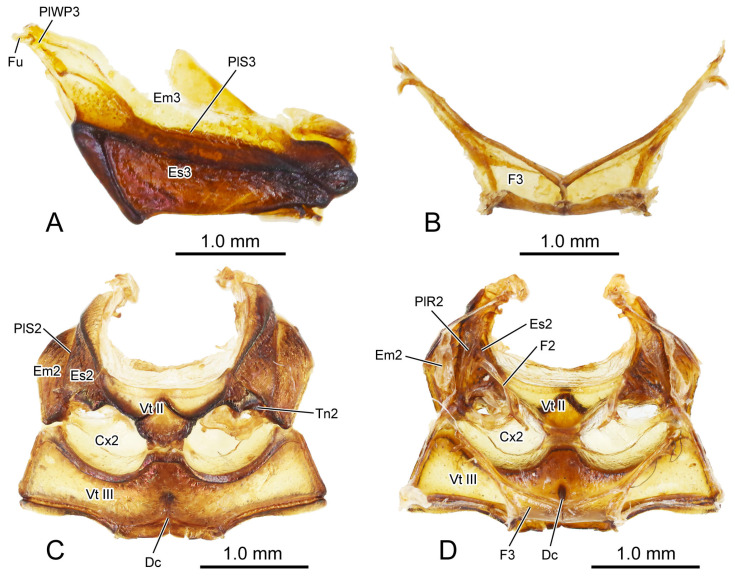
Digital photography of *Chrysolina sulcisollis*. (**A**) metathorax, lateral view; (**B**) metafurca, rear view; (**C**) meso- and metaventrites, ventral view; (**D**) meso- and metaventrite, dorsal view. Abbreviations: Cx2—mesocoxa; Dc—metathoracic discrimen; Em2/3—mes-/metepimeron; Es2/3—mes-/metanepisternum; F2/3—meso-/metafurca; Fu—fulcrum; PlWP3—pleural wing process of metathorax; PlR2—mesopleural ridge; PlS2/3—meso-/metapleural suture; Tn2—mesotrochantin; Vt II/III—meso-/metaventrite.

**Figure 3:**
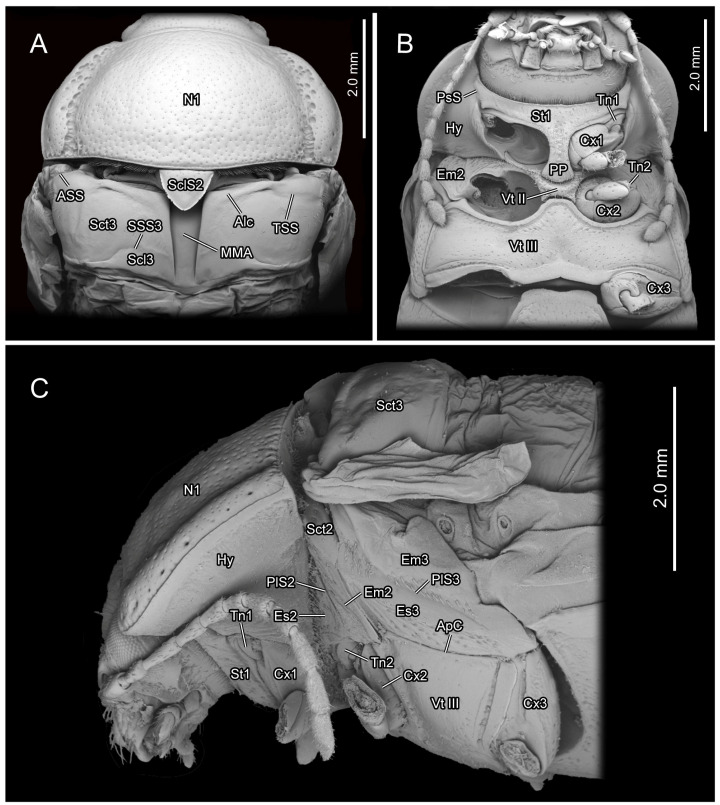
SEM photography of *Chrysolina sulcisollis*. (**A**) thorax without elytra, dorsal view; (**B**) thorax, ventral view; (**C**) thorax without elytra, lateral view. Abbreviations: Alc—alacrista; ApC—anapleural cleft; ASS—antero-lateral scutal suture; Cx1/2/3—pro-/meso-/metacoxal; Em2/3—mes-/metepimeron; Es2/3—mes-/metanepisternum; Hy—hypomeron; MMA—median membranous area; N1—pronotum; PlS2/3—meso-/metapleural suture; PP—prosternal process; PsP—notosternal suture; Scl3—metascutellum; SclS2—mesoscutellar shield; Sct2/3—meso-/metascutum; SSS3—scutoscutellar suture of metathorax; St1—prosternum; Tn1/2—pro-/mesotrochantin; TSS—transverse scutal suture; Vt II/III—meso-/metaventrite.

**Figure 4:**
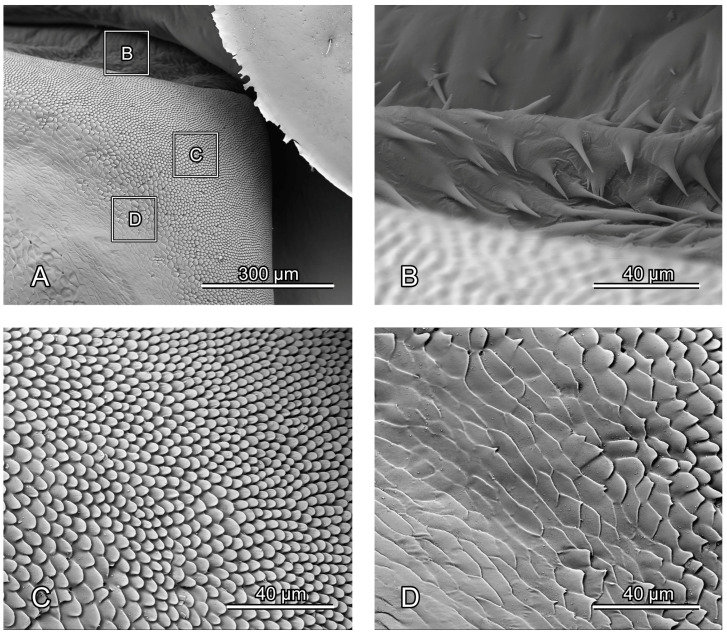
SEM photography of alacrista of *Chrysolina sulcisollis*. (**A**) alacrista; (**B**,**C**) setae on different parts of alacrista; (**D**) scales on lateral margin of alacrista.

**Figure 5:**
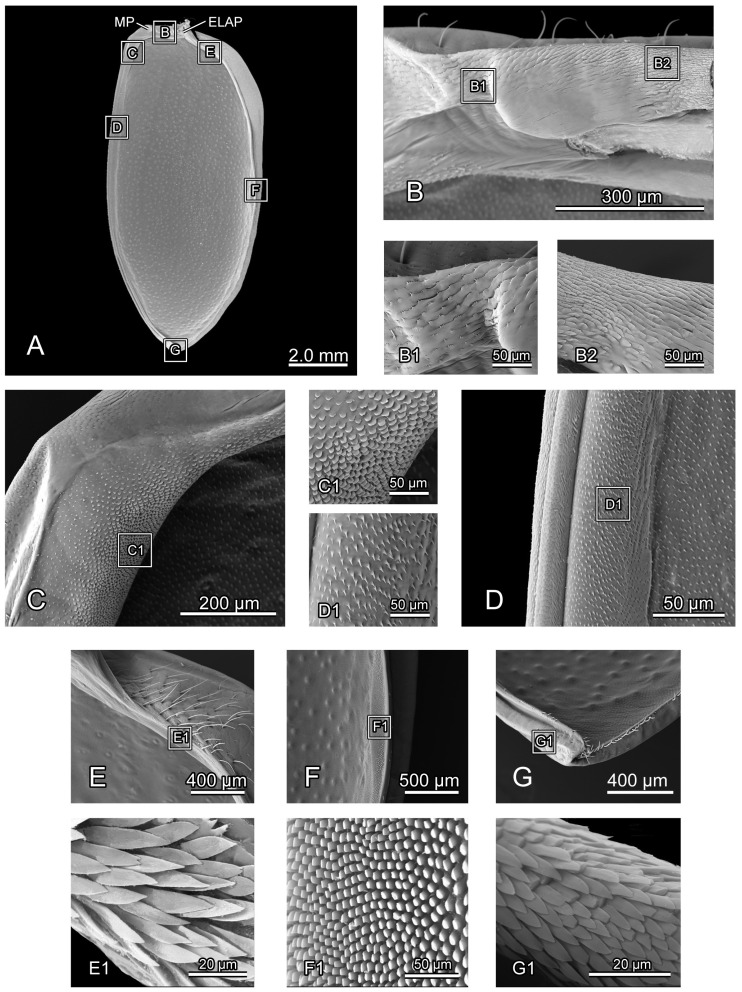
SEM photography of elytron of *Chrysolina sulcisollis*. (**A**) elytron, ventral view; (**B**) elytral base; (**B1**) michrotrichia on fold ridges in latero-anterior area of elytral base; (**B2**) wrinkles of elytral base; (**C**) anterior area of mesal suture; (**C1**) scales on antero-inner margin of mesal suture; (**D**) median area of mesal suture; (**D1**) setae on meso-inner region of mesal suture; (**E**) anterior area of epipleuron; (**E1**) setae on antero-inner region of the ridge; (**F**) median area of epipleuron. (**F1**) setae on meso-inner region of the ridge. (**G**) posterior area of elytron. (**G1**) setae on postero-lateral region of mesal suture. Abbreviations: ELAP—articulating process of elytron; MP—mesal process.

**Figure 6:**
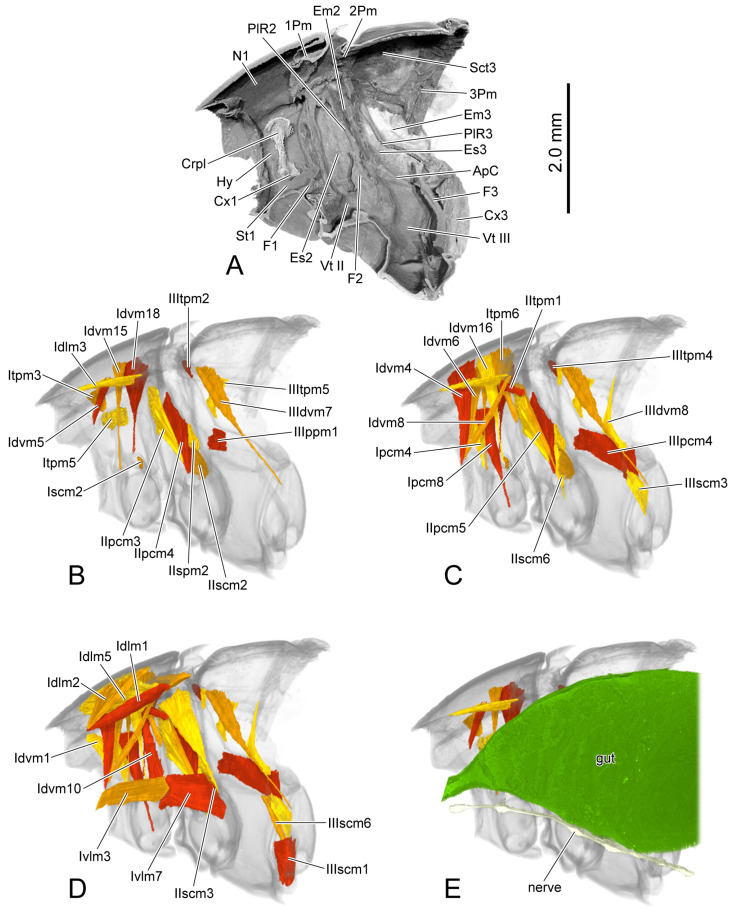
3D reconstruction of thoracic skeleton, musculature and other organs of *Chrysolina sulcisollis* in sagittal section. (**A**–**E**) indicate a gradual movement from the lateral position of the thorax to the proximal position. Abbreviations: 1/2/3Pm—pro-/meso-/metaphragma; ApC—anapleural cleft; Crpl—crytopleuron; Cx1/3—pro-/metacoxal; Em2/3—mes-/metepimeron; Es2/3—mes-/metanepisternum; F1/2/3—pro-/meso-/metafurca; Hy—hypomeron; N1—pronotum; PlR2/3—meso-/metapleural suture; Sct3—metascutum; St1—prosternum; Vt II/III—meso-/metaventrite.

The thoracic morphology was described based on observations from light microscopy (Figure 1 and Figure 2), SEM (Figure 3, Figure 4 and Figure 5), and 3D reconstruction (Figure 6).

#### 3.1.1. Prothoracic Skeleton

The well-developed and highly sclerotized prothorax is the largest segment of the thorax. Anteriorly, the prothorax articulates with the head through the cervical membrane, where lateral cervical sclerites are absent [28,29]. All prothoracic sclerites fuse together to form a rigid ring-like structure. Dense short fine setae are present along the anterior and posterior margins of the prothorax, with additional scattered short thick setae on the prosternum.

In the dorsal view, the width of the pronotum (N1: Figure 1A, Figure 3A,C and Figure 6A) is 2.2 times its length. The punctures scattered across the median area of the pronotum are small and shallow, while those on the lateral side are larger and deeper. The anterior margin of the pronotum slightly concaves posterad, forming a shallow wide depression that accommodates the occiput. The antero-lateral corner of the pronotum forms an obtuse angle and projects antero-mediad. Laterally, a groove delimits the thickened margin from the central panel of the pronotum. The posterior margin of the central panel of the pronotum slightly bends posterad. Ventrally, it connects with the bent anterad prophragma (1Pm: Figure 1E and Figure 6A).

In the ventral view of the prothorax, the bulged hypomeron (Hy: Figure 1B and Figure 3B,C) connects laterally with the thickened pronotal margin and occupies the lateral part of the sclerotic structure on the ventral side. The notosternal suture (PsS: Figure 1B) extends postero-laterad from the antero-lateral corner of the prosternum to the anterior 1/3 of the prothoracic ventral side, delimiting the T-shaped prosternum (St1: Figure 1B,C) from the lateral hypomera. The anterior portion of the prosternum is transversely slender, while the postero-median portion bears a long, trapezoid prosternal process (PP: Figure 1B and Figure 3B) that extends posterad between the paired procoxae. The prosternal process is narrow anteriorly, wide posteriorly and curved proximad laterally, with its posterior margin slightly bending posteriorly. The anterad bent protrochantin (Tn1: Figure 1B and Figure 3B) is nearly a flat trapezoid, with both the long anterior and short posterior margins bent anterad. Anteriorly, the protrochantin attaches to the postero-lateral margin of the anterior portion of the prosternum, and posteriorly it connects to the procoxal base. The slender crytopleuron (Crpl: Figure 6A) is situated near the lateral margin of the procoxal rim and extends dorsad along the hypomeron. Its dorsal portion broadens into a fan shape near the dorso-lateral margin of the pronotum. The short paired arms of the profurca (F1: Figure 6A) arise dorso-laterad separately from posterior margin of the prosternum, with each tip expanding into a posterad bent tray-like structure.

#### 3.1.2. Prothoracic Musculature

Idlm1 M. prophragma-occipitalis. O (=origin): antero-lateral area of ventral face of prophragma. I (=insertion): dorso-lateral area of occiput. Long triangular, narrowing towards occiput, straight.

Idlm2 M. pronoto-occipitalis. O: postero-median area of pronotum. I: dorso-lateral area of occiput. Long triangular, narrowing towards occiput, straight, broad, flat.

Idlm3 M. prophragma-cervicalis. O: lateral part of anterior margin of prophragma. I: dorso-lateral area of cervical membrane. Long triangular, narrowing towards cervical membrane, straight, slender.

Idlm5 M. pronoto-phragmalis anterior. O: antero-median area of pronotum. I: antero-lateral area of ventral face of prophragma. Irregular quadrilateral, original end broader than insertional end, straight, broad, flat.

Idvm1 M. cervico-occipitalis anterior. O: ventro-lateral area of ventral cervical membrane. I: dorso-lateral area of occipitale. Long triangular, narrowing towards occipitale, bent dorso-posterad.

Idvm4 M. pronoto-cervicalis lateralis. O: meso-lateral area of pronotum. I: postero-ventral area of occiput. Long triangular, narrowing towards occiput, straight.

Idvm5 M. pronoto-cervicalis anterior. O: meso-lateral area of pronotum. I: dorso-lateral area of cervical membrane. Approximate parallelogram, original end broader than insertional end, straight, slender.

Idvm6 M. pronoto-cervicalis medialis. O: meso-lateral area of pronotum. I: ventro-lateral area of ventral cervical membrane. Long triangular, narrowing towards cervical membrane, slightly bent postero-proximad, long.

Idvm8 M. prophragma-tentoralis. O: antero-lateral area of ventral face of prophragma. I: occipitale. Broad medially and narrowing towards both ends, slightly bent postero-laterad, long, slender.

Idvm10 M. profurca-phragmalis. O: dorso-lateral part of profurcal arm. I: antero-lateral area of ventral face of prophragma. Long triangular, narrowing towards prophragma, straight.

Idvm15 M. pronoto-trochantinocoxalis. O: postero-lateral area of pronotum. I: antero-proximal margin of procoxal rim. Long triangular, narrowing towards procoxal rim, straight, long, slender.

Idvm16 M. pronoto-coxalis anterior. O: postero-lateral area of pronotum. I: postero-lateral margin of procoxal rim. Long triangular, narrowing towards procoxal rim, straight, long.

Idvm18 M. pronoto-coxalis lateralis. O: postero-lateral area of pronotum. I: postero-lateral margin of procoxal rim. Long triangular, narrowing towards procoxal rim, straight, long.

Itpm3 M. pronoto-pleuralis anterior. O: antero-lateral area of pronotum. I: antero-dorsal area of lateral face of fan-shaped crytopleural dorsal portion. Triangular, narrowing towards crytopleuron, straight, short.

Itpm5 M. pronoto-apodemalis posterior. O: postero-lateral area of pronotum. I: postero-ventral area of lateral face of fan-shaped cryptopleural dorsal portion. Irregular quadrilateral, original end broader than insertional end, straight, short.

Itpm6 M. pronoto-intersegmentalis. O: postero-lateral area of pronotum. I: intersegmental membrane between pro- and mesothorax. Long triangular, narrowing towards intersegmental membrane, bent antero-laterad, long.

Ipcm4 M. propleuro-coxalis superior. O: antero-ventral area of proximal face of cryptopleural dorsal portion. I: antero-lateral margin of procoxal rim. Long triangular, narrowing towards procoxal rim, straight.

Ipcm8 M. propleuro-trochanteralis. O: postero-dorsal area of proximal face of cryptopleural dorsal portion. I: protrochanter. Long triangular, narrowing towards protrochanter, straight, long.

Ivlm3 M. profurca-tentorialis. O: dorsal area of anterior face of profurcal arm. I: ventro-median area of occipitale. Parallelogram, straight, long, broad.

Ivlm7 M. profurca-mesofurcalis. O: posterior face of profurcal arm. I: anterior face of mesofurcal arm. Irregular quadrilateral, original end narrower than insertional end, slightly bent dorso-proximad, large, flat.

Iscm2 M. profurca-coxalis posterior. O: ventro-lateral margin of profurcal arm. I: postero-lateral margin of procoxal rim. Long triangular, narrowing towards procoxal rim, straight, short.

#### 3.1.3. Mesothoracic Skeleton

The exposed part of the mesothoracic tergite from the dorsal view is triadius-shaped. The dense short white setae cover the mesoscutum, whereas the mesoscutellum (Scl2: Figure 1D,E) has no setae. The mesoscutum (Sct2: Figure 2C,D) occupies the anterior two branches and the central area of the triadius-shape. The small median process on the antero-lateral margin of the mesoscutum is the anterior notal wing process (AWP2: Figure 1E), and the inflated obtuse postero-lateral corner is the posterior notal wing process (PWP2: Figure 1D). The nearly equilaterally triangular posterior branch of the mesothorax in the dorsal view is the mesoscutellum, which is delimited from the anterior mesoscutum by the transverse scutoscutellar suture (SSS2: Figure 2D). The prominently raised triangular mesoscutellar shield (SclS2: Figure 1C and Figure 3A) aligns with the antero-proximal edge of the elytron when the elytra are closed. In the ventral view of the mesothoracic tergite, the axillary cord (AxC2: Figure 1E) runs along the scutoscutellar suture and the posterior margin of the mesoscutum, extending to a position behind the posterior notal process. The slender transverse mesophragma (2Pm: Figure 1F,G and Figure 6A) medially bends posterad and laterally links to the antero-proximal corners of the paired yoke plates.

The plicated mesopleuron is a nearly long triangle. The mesopleural suture (PlS2: Figure 2C and Figure 3C) extends from the antero-dorsal corner to the meso-ventral area of the mesopleuron, dividing it into the anterior mesanepisternum (Es2: Figure 2C,D, Figure 3C and Figure 6A) and the posterior mesepimeron (Em2: Figure 2C,D, Figure 3B,C and Figure 6A). The mesopleural suture invaginates inward, forming a mesopleural ridge (PlR2: Figure 2D and Figure 6A).

The flat T-shaped mesoventrite (Vt II: Figure 2C,D, Figure 3B and Figure 6A) is covered with thick setae. It is positioned anteriorly attaching to the posterior margin of the prothorax, with its antero-median part overlapped by the terminal portion of the prosternal process and its lateral margin connecting to the antero-ventral margin of the mesanepisternum. The bulged meso-posterior region of the mesoventrite is delineated by a medially anterad concaved margin. The small and anterad bent mesotrochantin (Tn2: Figure 2C and Figure 3B) antero-laterally attaches to the postero-lateral margin of the mesoventrite. The slender arm of the mesofurca (F2: Figure 2D and Figure 6A) extends dorso-laterad from the posterior margin of the mesoventrite, with its slightly expanded dorsal part positioned near the ventral part of the mesopleural ridge and bent antero-dorsad.

The elytron (Ely: Figure 1A) is long and nearly oval in shape (Figure 5A), with a length 2.17 times its width. From the dorsal view, circualar punctures are irregularly distributed across the discal region (Figure 1A), and the anterior margin of the elytra bears a slightly protruding humeral callus (HC: Figure 1A). From the ventral view, a sharp angulate lateral articulating process of elytron (ELAP: Figure 5A) extends antero-laterad, with its broad base positioned along the elytral base. The edge surrounding the discal region of the elytron is densely covered with setae or scales. The elytral base (Figure 5B) has dense wrinkles (Figure 5B2), with the fold ridges in the latero-anterior area bearing several michrotrichia (Figure 5B1). A prominent mesal process (MP: Figure 5A) is situated antero-proximally at the elytral base. The mesal suture is situated along the inner edge, forming the interlocking mechanism with the mesoscutellum and the elytron on the opposite side. The antero-proximal corner of the mesal suture protrudes antero-proximad and fits into the postero-lateral outline of the mesoscutellar shield. The mesal suture has obtuse scales along its antero-inner margin (Figure 5C,C1), spinous setae in the meso-inner region (Figure 5D,D1), and lanceolate setae along its postero-lateral margin (Figure 5G1). The epipleuron, located along the lateral edge, has a slender raised inner ridge. Bent setae are present on the anterior area of the epipeluron and along the lateral margin of the inner ridge (Figure 5E,G). The inner margin of the ridge has lanceolate setae in the anterior region (Figure 5E1) and nearly cylinder-shaped setae in the median region (Figure 5F,F1).

#### 3.1.4. Mesothoracic Musculature

IIdlm1 M. prophragma-mesopragmalis. O: meso-lateral region of ventral face of prophragma. I: meso-lateral region of mesophragma. Irregular quadrilateral, original end broader than insertional end, straight, large, flat.

IIdvm6 M. mesocoxa-subalaris. O: postero-lateral margin of mesocoxal rim. I: postero-lateral margin of mesonotum. Long triangular, narrowing towards mesocoxal rim, slightly bent postero-proximad, long, thick.

IItpm1 M. prophragma-mesanepisternalis. O: antero-lateral corner of ventral face of prophragma. I: antero-dorsal margin of mesanepisternum. Parallelogram, short, straight.

IIspm2 M. mesofurca-pleuralis. O: lateral margin of dorsal part of mesofurcal arm. I: ventral part of mesopleural ridge. Irregular quadrilateral, original end narrower than insertional end, slightly bent postero-laterad, flat, short.

IIpcm3 M. mesanepisterno-coxalis anterior. O: antero-dorsal area of mesanepisternum. I: antero-lateral margin of mesocoxal rim. Long triangular, narrowing towards mesocoxal rim, straight, long.

IIpcm4 M. mesanepisterno-coxalis posterior. O: posterior area of mesanepisternum. I: antero-lateral margin of mesocoxal rim. Approximate long triangular, narrowing towards mesocoxal rim, straight, long.

IIpcm5 M. mesanepisterno-trochanteralis. O: dorsal area of mesanepisternum. I: mesotrochanter. Long triangular, narrowing towards mesotrochanter, straight, long.

IIscm2 M. mesofurca-coxalis posterior. O: postero-lateral part of mesofurcal arm. I: postero-lateral margin of mesocoxal rim. Triangular, narrowing towards mesocoxal rim, straight, short.

IIscm3 M. mesofurca-coxalis posterior. O: postero-median part of mesofurcal arm. I: proximal margin of mesocoxal rim. Triangular, narrowing towards mesocoxal rim, slightly bent proximad, short.

IIscm6 M. mesofurca-trochanteralis. O: ventral face of dorsal part of mesofurcal arm. I: mesotrochanter. Long triangular, narrowing towards mesotrochanter, slightly bent antero-proximad, long.

#### 3.1.5. Metathoracic Skeleton

The metathorax is 4.31 times as long as its width. The metanotum is less sclerotized than the pro- and mesonotum. The intersegmental membrane between meso- and metatergite has sharp spines (Figure 4A,B). The metascutum (Sct3: Figure 1C,F,G, Figure 3A,C and Figure 6A) has a stripe-like median membranous area (MMA: Figure 1F,G and Figure 3A) that gradually narrows from anterior to posterior. The bulged alacrista (Alc: Figure 1C,F and Figure 3A) extends along the latero-anterior margin of the median depression to the antero-proximal margin of the metascutum. Dense oval-shaped setae are distributed on the alacrista (Figure 4A,C), while its lateral margin is covered by overlapping irregular scales (Figure 4A,D). The transverse scutal suture (TSS: Figure 1C,F,G and Figure 3A) extends latero-posterad from the distal end of the alacrista to the antero-lateral area of the metascutum. The antero-lateral scutal suture (ASS: Figure 1C,F,G and Figure 3A) extends along the antero-lateral margin of the metascutum, originating from the junction between the yoke plate and the metascutum, and delineates the long triangular anterior notal wing process (AWP3: Figure 1C) from the rest of the metascutum. Posteriorly, the obtuse posterior notal wing process (PWP3: Figure 1F,G) is positioned near the latero-posteral margin of the metascutum. The metascutellum (Scl3: Figure 1C,F,G and Figure 3A) is divided medially into a pair of semicircles by the median depression. It is separated from the anterior metascutum by scuto-scutellar suture (SSS3: Figure 1C,F,G and Figure 3A), which bends anterad. The narrow metaphragma (3Pm: Figure 6A) is located along the posterior margin of the metanotum and expands antero-ventrad.

The curved metapleural suture (PlS3: Figure 2A and Figure 3C) divides the irregularly quadrangular metapleuron into an antero-ventral metanepisternum (Es3: Figure 2A and Figure 3C) and a postero-dorsal metepimeron (Em3: Figure 2A and Figure 3C), and evaginates inward to form the metapleural ridge (PlR3: Figure 6A). The pleural wing process of the metathorax (PlWP3: Figure 2A) extends antero-dorsad from the antero-dorsal corner of the metanepisternum to articulate with the wing base. At its apex, it bifurcates, with the anterior branch forming the fulcrum (Fu: Figure 2A). The anterior margin of the metanepisternum has a bulged, highly sclerotized and narrow region. Ventrally the metapleuron is separated from the metaventrite by the anapleural cleft (ApC: Figure 3C).

The transverse metaventrite (Vt III: Figure 2C,D, Figure 3B,C and Figure 6A) is covered by scattered short setae. Both its anterior and posterior margins have a pair of depressions that, respectively, accommodate the meso- and metacoxae. The antero-median process and antero-lateral corner, respectively, connect to the posterior margin of the mesoventrite and the ventro-posterior corners of the mesepimeron. Additionally, its postero-median margin and postero-lateral corners connect to the first abdominal sternite. The arm of the metafurca (F3: Figure 2B and Figure 6A) extends dorso-laterad from the posterior margin of the metaventrite, gradually tapering distally, and terminates a dorsoventrally forked apex. The metathoracic discrimen (Dc: Figure 2C,D) lies along the midline, extending from the center to the posterior margin of the metaventrite, where it supports the posterior metafurca.

#### 3.1.6. Metathoracic Musculature

IIIdvm7 M. metanoto-trochanteralis. O: antero-lateral area of metanotum. I: metatrochanter. Long triangular, narrowing towards metatrochanter, slightly bent antero-proximad, long.

IIIdvm8 M. metafurca-phragmalis. O: apex of dorsal branch of metafurcal arm. I: latero-ventral area of anterior face of metaphragma. Long triangular, narrowing towards metascutum, slender, bent ventro-laterad.

IIItpm2 M. metapleural-prealaris. O: apex of metapleural ridge. I: lateral area of mesophragma. Long triangular, narrowing towards metapleural ridge, straight, short.

IIItpm4 M. mesonoto-pleuralis anterior. O: dorsal part of metapleual ridge. I: antero-lateral area of metanotum. Rectangular, slightly bent proximad, short, slender.

IIItpm5 M. metanoto-pleuralis medialis. O: dorso-median part of metapleural ridge. I: latero-median margin of metascutum. Irregular quadrilateral, original end broader than insertional end, bent anterad.

IIIppm1 M. metatransanapleuralis. O: median part of metapleural ridge. I: antero-lateral margin of metaventrite. Irregular quadrilateral, original end narrower than insertional end, straight, thick, flat.

IIIpcm4 M. metanepisterno-coxalis posterior. O: postero-ventral area of metanepisternum, beneath metapleural ridge. I: antero-lateral margin of metacoxal rim. Approximately triangular, narrowing towards metacoxal rim, straight, large.

IIIscm1 M. metafurca-coxalis anterior. O: proximo-ventral part of metafurcal arm. I: antero-proximal margin of metacoxal rim. Flat triangular, narrowing towards metacoxal rim, straight, large, flat.

IIIscm3 M. metafurca-coxalis medialis. O: postero-median part of metafurcal arm. I: meso-proximal margin of metacoxal rim. Irregular quadrilateral, original end narrower than insertional end, straight.

IIIscm6 M. metafurca-trochanteralis. O: latero-ventral part of metafurcal arm. I: metatrochanter. Long triangular, narrowing towards metatrochanter, straight, long, slender.

#### 3.1.7. Digestive System and Nervous System

The gut (gut: Figure 6E) is narrow at the anterior end of the thorax and gradually enlarges posteriorly, occupying nearly the entire thoracic cavity. Beneath the gut lies a slender nerve cord (nerve: Figure 6E), along which several enlarged ganglia are distributed.

### 3.2. Chrysolina (Euchrysolina) virgata (Motschulsky, 1860)

**Figure 7:**
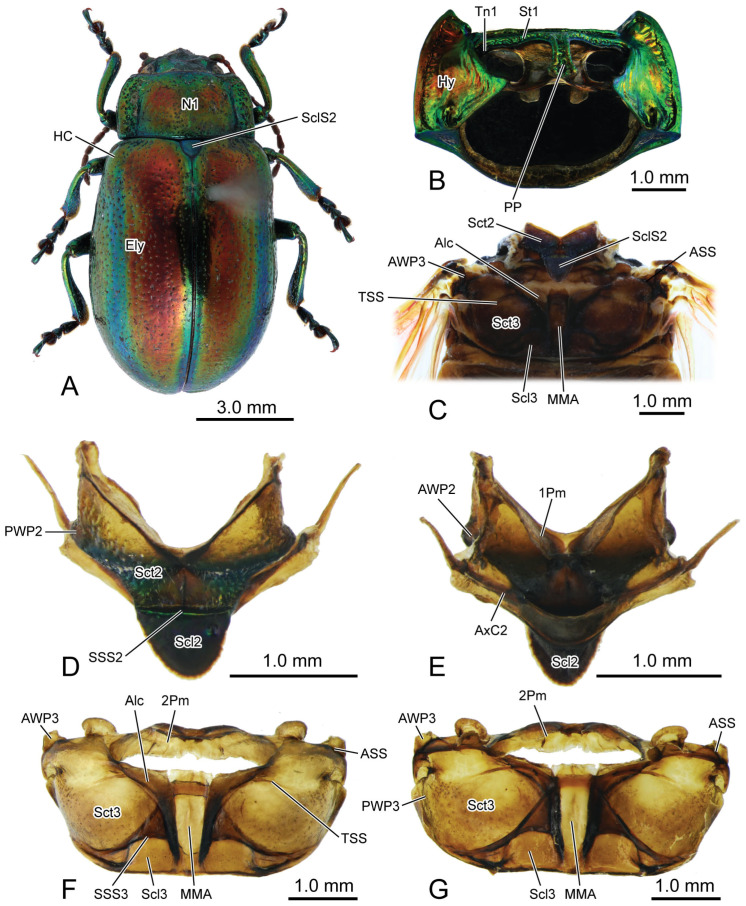
Digital photography of *Chrysolina virgata*. (**A**) habitus, dorsal view; (**B**) prothorax, ventral view; (**C**) pterothorax, dorsal view; (**D**) mesothoracic tergite, dorsal view; (**E**) mesothoracic tergite, ventral view; (**F**) metathoracic tergite, dorsal view; (**G**) metathoracic tergite, ventral view. Abbreviations: 1/2Pm—pro-/mesophragma; Alc—alacrista; ASS—antero-lateral scutal suture; AWP2/3—anterior notal wing process of meso-/metathorax; AxC2—axillary cord of mesothorax; Ely—elytron; HC—humeral callus; Hy—hypomeron; MMA—median membranous area; N1—pronotum; PP—prosternal process; PsS—notosternal suture; PWP2/3—posterior notal wing process of meso-/metathorax; Scl2/3—meso-/metascutellum; SclS2—mesoscutellar shield; Sct2/3—meso-/metascutum; SSS2/3—scutoscutellar suture of meso-/metathorax; St1—prosternum; Tn1—protrochantin; TSS—transverse scutal suture.

**Figure 8:**
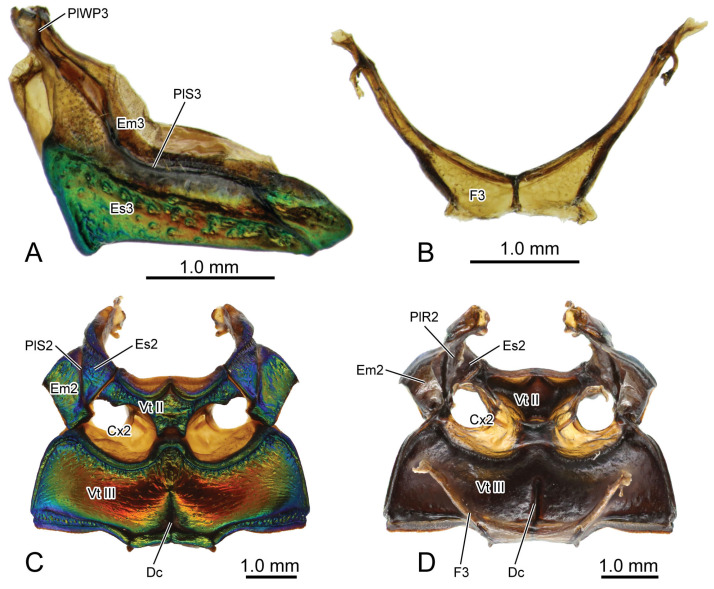
Digital photography of *Chrysolina virgata*. (**A**) metathorax, lateral view; (**B**) metafurca, rear view; (**C**) meso- and metaventrites, ventral view; (**D**) meso- and metaventrite, dorsal view. Abbreviations: Cx2—mesocoxa; Dc—metathoracic discrimen; Em2/3—mes-/metepimeron; Es2/3—mes-/metanepisternum; F3—metafurca; Fu—fulcrum; PlWP3—pleural wing process of metathorax; PlR2—mesopleural ridge; PlS2/3—meso-/metapleural suture; Tn2—mesotrochantin; Vt II/III—meso-/metaventrite.

**Figure 9:**
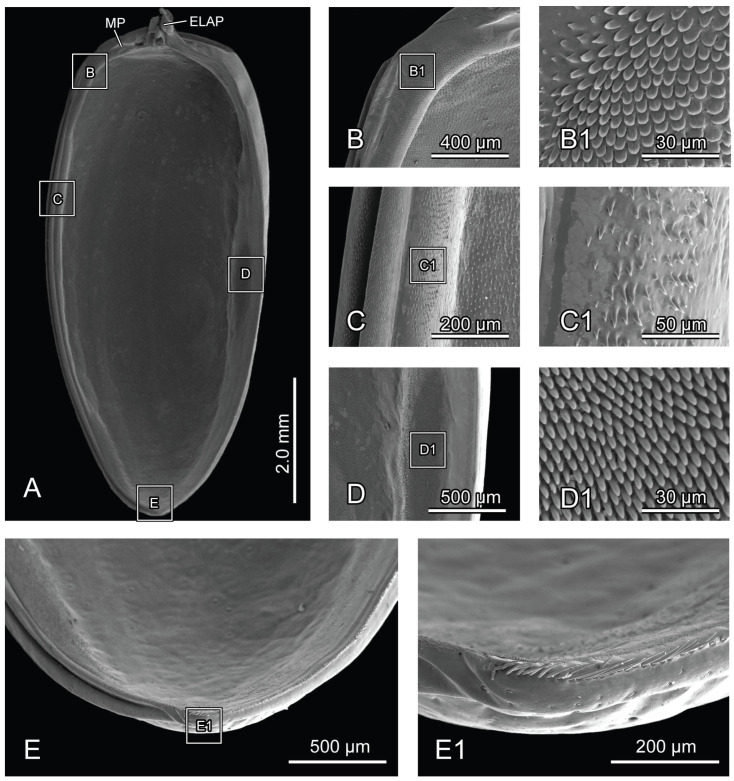
SEM photography of elytron of *Chrysolina virgata*. (**A**) elytron, ventral view; (**B**) anterior area of mesal suture; (**B1**) scales on antero-inner margin of mesal suture; (**C**) median area of mesal suture; (**C1**) setae on meso-inner region of mesal suture; (**D**) median area of epipleuron; (**D1**) setae on meso-inner region of ridge; (**E**) posterior area of elytron; (**E1**) setae on postero-lateral region of ridge. Abbreviations: ELAP—articulating process of elytron; MP—mesal process.

**Figure 10:**
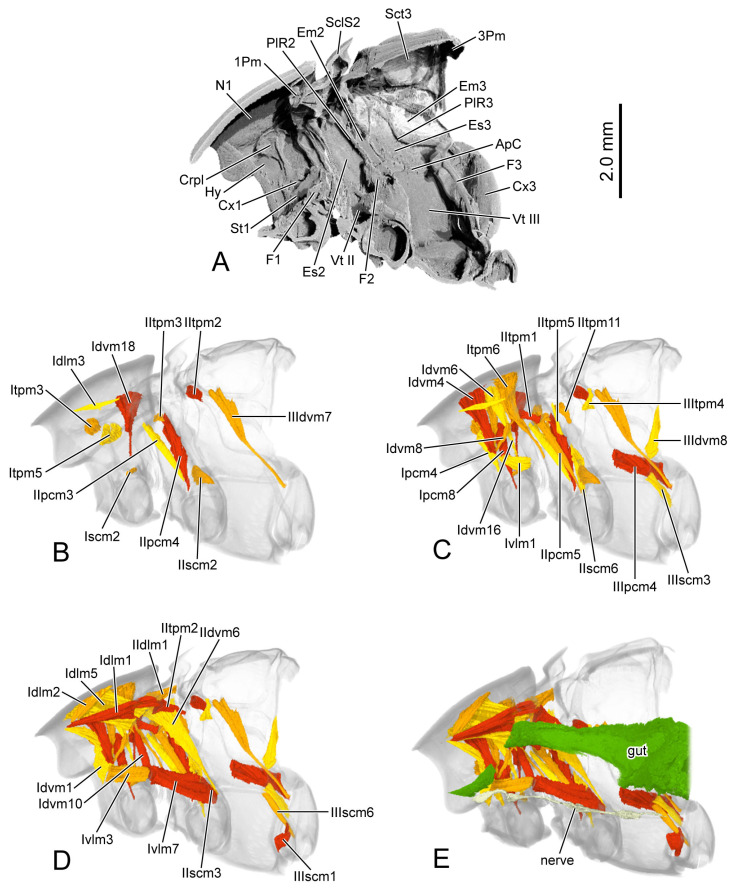
3D reconstruction of thoracic skeleton, musculature and other organs of *Chrysolina virgata* in sagittal section. (**A**–**E**) indicate a gradual movement from the lateral position of the thorax to the proximal position. Abbreviations: 1/2/3Pm—pro-/meso-/metaphragma; ApC—anapleural cleft; Crpl—crytopleuron; Cx1/3—pro-/metacoxal; Em2/3—mes-/metepimeron; Es2/3—mes-/metanepisternum; F1/2/3—pro-/meso-/metafurca; Hy—hypomeron; N1—pronotum; PlR2/3—meso-/metapleural suture; Sct3—metascutum; St1—prosternum; Vt II/III—meso-/metaventrite.

The thoracic morphology was described based on observations from light microscopy (Figure 7 and Figure 8), SEM (Figure 9), and 3D reconstruction (Figure 10). Only the thoracic morphological structures that differ from those of *C. sulicollis* are described here.

#### 3.2.1. Prothoracic Skeleton

In the dorsal view of the prothorax, the pronotum (N1: Figure 7A) exhibits a smaller width-to-length ratio of 1.68. The punctures scattered across the dorsal surface of the pronotum are larger and more sparsely distributed. The depression along the anterior margin of the pronotum has sharply angled corners, whereas, in *C. sulcicollis*, the corners are smoothly rounded. The posterior margin of the pronotum bends posterad more prominently. The anterior half of the prosternum and the posterior prosternal process are slenderer (St1, PP: Figure 7B).

#### 3.2.2. Prothoracic Musculature

The prothorax lacks Idvm5 but retains Ivlm1.

Idlm2: Flat triangular, narrowing towards occiput, straight, flat.

Idlm3: Broad medially and narrowing towards both ends, straight, slender.

Idvm6: Long triangular, narrowing towards cervical membrane, slightly bent postero-laterad, long.

Idvm16: Long triangular, narrowing towards procoxal rim, bent postero-laterad, long.

Itpm3: Trapezoid, original end broader than insertional end, straight, short.

Ivlm1 M. profurca-cervicalis. O: latero-ventral area of anterior face of profurcal arm. I: ventral cervical membrane. Approximately triangular, narrowing towards cervical membrane, straight.

#### 3.2.3. Mesothoracic Skeleton

The posterior margin of the mesoscutellum is more rounded. The mesophragma (2Pm: Figure 7F,G) is broader, with a shorter median part that bends posterad. The elongated elytron has a length 2.23 times its width. The lateral articulating process of elytron (ELAP: Figure 9A) extends anterad. The smoother anterior corner of the mesal suture and the mesal process of the elytral base (MP: Figure 9A) do not protrude prominently. The setae in the meso-inner region of the epipleuron are rodlike with the obtuse tips (Figure 9D1).

#### 3.2.4. Mesothoracic Musculature

The mesothorax lacks IIspm2 but retains IItpm2, 3, 5 and 11.

IItpm2 M. mesopleura-praealaris. O: dorso-posterior area of mesepimeron. I: ventro-lateral area of dorsal face of prophragma. Long triangular, narrowing towards mesepimeron, straight, long.

IItpm3 M. mesonoto-basalaris. O: dorso-median area of mesanepisternum. I: antero-lateral margin of mesonotum (mesobasalare). Triangular, narrowing towards mesanepisternum, straight, short.

IItpm5 M. mesonoto-pleuralis medialis. O: dorso-anterior area of mesopleural ridge. I: latero-median margin of mesonotum. Long triangular, narrowing towards mesonotum, straight, slender.

IItpm11 M. mesopleura-subalaris. O: dorso-posterior area of mesepimeron. I: postero-lateral margin of mesonotum. Irregular quadrilateral, original end broader than insertional end, straight, short.

IIscm3: Irregular quadrilateral, original end broader than insertional end, straight.

#### 3.2.5. Metathoracic Skeleton

The metanotum is longer and narrower, with the length 2.21 times its width. The sclerotized region along the anterior margin of the metanepisternum is slender (Figure 8A).

#### 3.2.6. Metathoracic Musculature

The metathorax lacks IIItpm5 and IIIppm1.

IIIdvm8: Irregular quadrilateral, original end narrower than insertional end, straight, broad.

IIItpm4: Irregular quadrilateral, original end broader than insertional end, curved.

IIIscm1: Irregular quadrilateral, original end broader than insertional end, bent ventro-proximad, flat.

#### 3.2.7. Digestive System and Nervous System

The gut (gut: Figure 10E) within the thorax is relatively narrow, gradually thickening from anterior to posterior and forming a distinct bend in the posterior region of the prothorax. (The interrupted section in the anterior part of the gut probably comes from specimen preservation issues or limitations in micro-CT scan quality.)

## 4. Discussion

### 4.1. Comparison of the Two Flightless Chrysolina Species

The skeletal morphology of the two species differs only in shape. The differences in the pro- and mesothorax are more pronounced than those in the metathorax. The most notable muscular difference (Table 1) lies in the tergo-pleural muscles of the mesothorax that connect to the basal sclerite of the elytron: *C. sulcisollis* has only one muscle, IItpm1, while *C. virgata* possesses five muscles—IItpm1, 3, 5 and 11. In addition, *C. sulcisollis* uniquely has the muscles Idvm5 and 15, IIspm2, IIItpm5 and IIIppm1, whereas *C. virgata* has the muscle Ivlm1. The other differences are reflected in their planar geometry and curvature. It is evident that *C. sulcisollis* has fewer tergo-pleural muscles functioning as the direct wing muscles to control elytral movement—a feature likely linked to its permanently locked elytra, which helps conserve water by providing a mechanical barrier that reduces direct water loss from the abdomen and spiracles [30]. This phenomenon might resemble the tightly fitted elytra found in *Elytrogona*, a flightless leaf beetle genus inhabiting the tropical regions of the Americas [16]. The morphological differences in the elytra between the two species, including the structure of elytral base, the anterior corner of the mesal suture, and the setae on the meso-inner region of epipleuron, might also contribute to this adaptation. Furthermore, large-bodied *C. sulcisollis* has more muscles that contribute to stabilizing body structures, particularly IIspm2 and IIIppm1. The observed difference in gut size might be attributed to *C. sulcisollis* having recently ingested food, whereas *C. virgata* had not. The functional significance of other morphological differences remains to be explored further.

### 4.2. Comparison with Other Flight-Capable Beetles

Beutel and Haas conducted a detailed examination of the endo- and exoskeletal structures in *Leptinotarsa decemlineata*, a flight-capable species used as a representative of Chrysomelidae, as part of their morphological analysis to construct a phylogenetic tree of Coleoptera [31]. A comparison of the thoracic sclerites between the two flightless beetles and *L. decemlineata* revealed no substantial differences. Some morphological structures, such as pronotum and elytra, exhibit considerable variation within the genus, making it difficult to assess their association with the loss of flight capability [16,17,18]. In addition, we referred to the comprehensive description of the coleopteran skeletal system by Larsen and Matsuda, which notes that lateral cervical sclerites are commonly present in Polyphaga except Tenebrionidae and Curculionidae [28,29]. In contrast, these sclerites are absent in the two flightless beetles. It remains unclear whether this absence is associated with the loss of flight ability.

We compared the thoracic musculature of the two flightless beetles with that of six flight-capable species *Cassida viridis*, *Lilioceris lilii*, *Donacia versicolorea*, *Donacia* sp., *Aulacophora foveicollis* (synonym: *Rhaphidopalpa femoralis*) and *Melasoma populi* also from Chrysomelidae (Table 1) [28,31,32]. The muscle nomenclature used by Larsen has already been homologized with the system used in this study [10,11,12]. Meanwhile, we also listed the functions of most thoracic muscles based on prior hypotheses [33,34,35]. Compared to the flight-capable species, which typically have 16 to 18 prothoracic muscles, *C. sulcisollis* and *C. virgata* possess more, with 21 and 20 muscles, respectively (Muscles of uncertain identity were not included in the count). The most notable difference is that the flightless beetles possess four muscles—Idvm4, 5, 18 and Itpm5—that are absent in all flight-capable species. These muscles are associated with head or leg movements, respectively.

The other chrysomelid beetles possess 14 to 15 mesothoracic muscles, whereas *C. sulcisollis* has only 10. *C. sulcisollis* shows a marked reduction in the direct wing muscles controlling elytral movement, retaining only IItpm1. In contrast, *C. virgata* has the highest number of tergo-pleural muscles, with a total of 6, whereas other flight-capable chrysomelid beetles have only 3 to 4. Otherwise, the flight-capable beetles have more indirect flight muscles including IIdlm2, IIdvm4 or 5, while the two flightless species exclusively have another direct flight muscle IIdvm6.

There are significant differences in the metathoracic musculature between the flight-capable and the two flightless species. Overall, the flight-capable beetles possess a markedly greater number of metathoracic muscles, with a total of 18 to 20, compared to 10 in *C. sulcisollis* and 8 in *C. virgata*. The primary indirect flight muscles that generate power for hindwing flight—the dorsal longitudinal muscle IIIdlm1 and the dorso-ventral muscles IIIdvm1–5—are completely absent in the two flightless species. The tergo-pleural muscles as the direct flight muscles, which connect the wing base sclerites and adjust the direction of wing movement, are limited to 3 in *C. sulcisollis*—IIItpm2, 4 and 5—and 2 in *C. virgata*—IIItpm2 and 4, whereas the flight-capable species, respectively, have 5—IIItpm2, 3, 7, 9 and 10. Additionally, all flight-capable species have the muscles IIIdvm6 and IIIspm1, which might be linked to flight capability through their connections to the wing base sclerites. These muscles are entirely absent in the two flightless species.

Besides the significant reduction in metathoracic muscles being associated with the loss of flight, the two flightless species of *Chrysolina* might have enhanced mobility in their neck, due to the exposure of more cuticular membranes could enhance flexibility. The absence of lateral cervical sclerites and the presence of muscles Idvm4, 5 and Itpm5 could permit a greater range of head movement to achieve a broader field of vision, thereby improving their ability to locate food, evade predators, and gain a competitive advantage. In addition, the mesothorax of *C. virgata* contains more direct flight muscles, which probably suggests that its elytra serves some uniquely significant function.

### 4.3. Comparison with Other Flightless Beetles

The anatomical studies indicate that the morphological structures related to flight have undergone degeneration in the thorax of all flightless beetles. The most notable feature is the substantial reduction in both indirect and direct flight muscles in the metathorax [9,10,11,12,13,14]. Both the meso- and metathoracic skeletal systems of the troglobiontic beetle *Troglocharinus ferreri* are strongly reduced. The triangular scutellar shield is the only distinctly developed structure in the mesothorax. The metanotum lacks recognizable sclerite subdivisions and wing-related elements. Additionally, the mesothoracic musculature is limited to just six muscles [11]. The three flightless weevils show profound modifications of the metathorax, including sclerite fusion, reduced length, and the loss of certain small structures [9]. The metathorax of the cave-dwelling specialist *Sinaphaenops wangorum* has shortened metanotum and metaventrite, along with a complete reduction in wing base sclerites [10]. The skeletal structures related to flight of the metathorax are still preserved in the two flightless species of Staphylinidae [12,13]. The thoracic skeletal structure of the flightless *Callosobruchus maculatus* remains largely unchanged, though several muscles are absent in the prothorax [14]. Taxonomic findings indicate that among flightless leaf beetles, species of *Elytrogona* and *Convexocoleus* exhibit an extremely convex body, fused medial elytral margins, a fused and membranous meso- and metanotum, and a narrowed metasternum [16,18]. In contrast, species of *Suinzona* lack humeral calli [20]. These morphological features might be associated with the loss of flight capability. However, *Stoiba* species with brachypterous wings display the same morphological characteristics as those with fully developed wings [17]. The extent of degeneration in other flight-related structures might indicate how early or late flight loss occurred during the course of evolution, or the intensity of evolutionary pressure. Otherwise, some of these flightless beetles have evolved a range of specialized structures to adapt to their unique habitats, such as caves [10], subterrane [11], or myrmecophilous environment [12], and particular survival strategy, such as thanatosis defense [9]. Despite the reduction in flight-related muscles, the skeletal structures of the pterothorax of the two flightless *Chrysolina* species remain intact with humeral calli on the elytra (HC: Figure 1A and Figure 7A), similar to those observed in flightless species of *Stoiba*. Comparative studies of the evolutionary process of the two flightless *Chrysolina* species with other flightless beetles require further data, including a comprehensive examination of habitat, functional compensation, and other relevant factors.

## Figures and Tables

**Table 1:** Thoracic musculature of *Cassida viridis* (abbreviated to Cv1), *Lilioceris lilii* (Ll), *Donacia versicolorea* (Dv), *Donacia* sp. (Ds), *Aulacophora foveicollis* (Af), *Melasoma populi* (Mp), *Chrysolina sulcisollis* (Cs) and *Chrysolina virgata* (Cv2) and the muscular functions (present with “+” or muscular name in green, absent with “-” in pink, uncertain with “?” in yellow).

Muscles	Cv1	Ll	Dv	Ds	Af	Mp	Cs	Cv2	Functions
Prothorax									
Idlm1	+	+	+	+	1	+	+	+	Head levator/abductor/rotator/lateral translator
Idlm2	+	+	+	+	4	+	+	+	Control head movement
Idlm3	-	+	+	+	-	+	+	+	Control head or neck movement
Idlm5	+	+	+	+	2	+	+	+	Pronotum retractor
Idvm1	-	-	-	-	8	-	+	+	Control head movement
Idvm2	+	+	+	+	-	+	-	-	
Idvm3	+	+	+	+	-	+	-	-	
Idvm4	-	-	-	-	-	-	+	+	Control head or neck movement
Idvm5	-	-	-	-	-	-	+	+	
Idvm6	+	+	+	+	-	+	+	+	Occipital process levator
Idvm8	-	+	+	+	10	-	+	-	Control head movement
Idvm10	+	+	+	+	-	+	+	+	Not been explicitly labeled
Idvm12	-	-	-	-	11	-	-	-	
Idvm13	-	+	+	+	-	+	-	-	Leg muscles
Idvm15	-	-	-	-	13	-	+	-	
Idvm16	+	+	+	+	14	+	+	+	
Idvm17	+	+	+	+	-	+	-	-	
Idvm18	-	-	-	-	-	-	+	+	
Itpm3	+?	+?	+?	+?	12	+?	+	+	Occipital process levator/adductor/rotator
Itpm5	-	-	-	-	-	-	+	+	Occipital process depressor/protractor
Itpm6	+	+	+	+	3	+	+	+	Leg muscles
Ipcm4	+	+	+	+	16	+	+	+	
Ipcm8	+	+	+	+	17	+	+	+	Not been explicitly labeled
Ivlm1	+	+	+	+	7	+	-	+	Occipital process levator/adductor/rotator
Ivlm3	+	+	+	+	5, 6	+	+	+	Head depressor
Ivlm7	+	+	+	+	20	+	+	+	Not been explicitly labeled
Iscm2	+	-	-	-	15	+	+	+	Leg muscle
Mesothorax									
IIdlm1	+	+	+	+	18	+	+	+	Indirect wing muscle
IIdlm2	+	+	+	+	19	+	-	-	
IIdvm4	+?	+?	+?	+?	28	+?	-	-	Indirect wing muscle, leg muscle
IIdvm5	+	+	+	+	-	+	-	-	
IIdvm6	-	-	-	-	-	-	+	+	Direct wing muscle, leg muscle
IItpm1	-	-	-	-	-	-	+	+	Direct wing muscles
IItpm2	+	+	+	+	21	+	-	+	
IItpm3	-	-	-	-	-	-	-	+	
IItpm4	-	-	-	-	22	-	-	-	
IItpm5	-	-	-	-	-	-	-	+	
IItpm7	+	+	+	+	-	+	-	-	
IItpm9	+	+	+	+	-	+	-	-	
IItpm10	+	+	+	+?	24, 25	+	-	+	
IItpm11	-	-	-	-	23	-	-	+	
IIspm2	+	+	+	+	26	+	+	-	Not been explicitly labeled
IIpcm2	-	-	-	-	31	-	-	-	Direct wing muscles, leg muscles
IIpcm3	+	+	+	+	30	+	+	+	
IIpcm4	+	+	+	+	-	+	+	+	Leg muscle
IIpcm5	-	+	-	-	32?	-	+	+	Direct wing muscle, leg muscle
IIpcm6	+	-	+	+	-	+	-	-	Leg muscle
IIvlm3	+	+	+	+	37	+	-	-	Not been explicitly labeled
IIscm1	+	+	+	+	27	+	-	-	Leg muscles
IIscm2	+	+	+	+	29	+	+	+	
IIscm3	-	-	-	-	-	-	+	+	
IIscm6	+	+	+	+	33	+	+	+	
Metathorax									
IIIdlm1	+	+	+	+	35	+	-	-	Indirect wing muscle
IIIdlm2	+	+	+	+	36	+	-	-	
IIIdvm1	+	+	+	+	38	+	-	-	
IIIdvm2	+	+	+	+	39	+	-	-	Indirect wing muscles, leg muscles
IIIdvm3	-	-	-	-	50	-	-	-	
IIIdvm4	-	-	-	-	52	-	-	-	
IIIdvm5	+	+	+	+	-	+	-	-	
IIIdvm6	+	+	+	+	53	+	-	-	Direct wing muscle, leg muscle
IIIdvm7	-	-	-	-	-	-	+	+	Indirect wing muscle, leg muscle
IIIdvm8	+	+	+	+	-	+	+	+	Not been explicitly labeled
IIItpm2	+	+	+	+	41	+	+	+	Direct wing muscles
IIItpm3	+	+	+	+	40	+	-	-	
IIItpm4	-	-	-	-	-	-	+	+	
IIItpm5	-	-	-	-	-	-	+	-	
IIItpm7	+	+	+	+	42–44	+	-	-	
IIItpm9	+	+	+	+	45	+	-	-	
IIItpm10	+	+	+	+	46	+	-	-	
IIIppm1	-	-	-	-	49	+	+	-	Not been explicitly labeled
IIIspm1	+	+	+	+	47	+	-	-	Direct wing muscle
IIIspm2	-	-	-	-	48	-	-	-	Not been explicitly labeled
IIIpcm3	+	-	+	-	55	-	-	-	Direct wing muscle, leg muscle
IIIpcm4	+	+	+	+	56	+	+	+	Leg muscles
IIIpcm6	-	-	-	-	57	-	-	-	
IIIscm1	+	+	+	+	51	+	+	+	
IIIscm2	+	+	+	+	54	+	-	-	
IIIscm3	-	-	-	-	-	-	+	+	
IIIscm4	+	+	+	+	-	+	-	-	
IIIscm6	+	+	+	+	-	+	+	+	

## Data Availability

The data presented in this study are available on request from the corresponding author due to privacy and legal reason.
